# Cylindrical coordinate‐based TG‐43U1 parameters for dose calculation around elongated brachytherapy sources

**DOI:** 10.1120/jacmp.v9i2.2760

**Published:** 2008-04-16

**Authors:** Shahid B. Awan, Sharifeh A. Dini, Manzoor Hussain, David Soleimani‐Meigooni, Ali S. Meigooni

**Affiliations:** ^1^ University of Kentucky Department of Radiation Medicine Lexington Kentucky U.S.A.

**Keywords:** RadioCoil, ${}^{103}Pd$, TG‐43U1, cylindrical coordinate system, polar coordinate system

## Abstract

In 2001, the use of cylindrical coordinates was demonstrated to be more suitable than was the use of polar coordinates for accurate computer calculations during treatment planning for I192r intravascular brachytherapy sources. In the present work, we investigated the applicability of cylindrical coordinate–based TG‐43U1 parameters for dosimetric evaluation and dose calculations for RadioCoil ${}^{103}Pd$ sources (RadioMed Corporation, Tyngsboro, MA) 1.0‐cm to 6.0‐cm long. For brevity, only the results for sources 1.0‐cm, 3.0‐cm, and 5.0‐cm long are presented here. Dosimetric characteristics of RadioCoil ${}^{103}Pd$ sources were calculated in liquid water using the Monte Carlo simulation technique. To demonstrate the suitability of this methodology, the Monte Carlo–simulated dose profiles for a RadioCoil ${}^{103}Pd$ source 5.0‐cm long at radial distances of 0.5 cm, 0.9 cm, and 1.25 cm were compared with calculated data using TG‐43U1 parameters in the polar and cylindrical coordinate systems. In addition, we also used a source 1.0‐cm long parameterized using cylindrical coordinates to investigate the application of a linear segmented source (LSS) model originally introduced by our group. The results indicate that, for dose calculation around elongated brachytherapy sources, cylindrical coordinate–based TG‐43U1 parameters more accurately represent the dose distribution around an elongated source than the polar coordinate–based parameters. In addition, the LSS model, in conjunction with the cylindrical coordinate–based parameters for a source 1.0‐cm long, can be used to replicate the dose distribution around any integral source length. This process eliminates the need to collect and enter data for multiple source lengths into treatment planning systems.

PACS number: 87.66.Jj

## I. INTRODUCTION

Since the late 1990s, brachytherapy treatments have been widely expanded into management of various tumor sites such as prostate, breast, and cervix. The success of this treatment modality is partly attributable to advances in the dosimetric evaluation of brachytherapy sources and treatment procedures.

The original and updated recommendations of Task Group 43 (TG‐43 and TG‐43U1 respectively) of the American Association of Physicists in Medicine (AAPM) are the foundation of current brachytherapy source dosimetry procedures.^(^ ^1^ ^,^ ^2^ ^)^ The TG‐43 protocols have been extensively used to determine the dosimetric characteristics of various source types and models with active lengths of 1.0 cm or less.^(^ ^3^ ^–^ ^9^ ^)^ The original TG‐43 protocol, introduced in 1995, was based on recommendations of the Interstitial Collaborative Working Group^(10)^ and provided limited published dosimetric data. Their data included ^125^I [models 6711 and 6702 (Amersham/Oncura, Plymouth Meeting, PA)],${}^{103}Pd$ [model 200 (Theragenics Corporation, Norcross, GA)], and ${}^{192}Ir$ (Best Industries, Springfield, VA) sources.^(1)^ An update to the TG‐43 protocol (TG‐43U1)^(2)^ was introduced in 2004 as a result of developments in the technology, discovery of some shortcomings in the original protocol, availability of more brachytherapy source dosimetry data, and the introduction of new source models. Per the TG‐43U1 protocol, the two‐dimensional (2D) anisotropy function for all brachytherapy sources should, at a minimum, be reported for radial distances r={0.5,1,2,3,5,and 7 cm} for ^125^I and {0.5, 1, 2, 3, and 5 cm} for ${}^{103}Pd$, from θ={0 to 90 degrees in 10‐degree increments}. In addition, the recommendations state that, to minimize extrapolation, F(r,θ) data should be determined over the widest reasonably achievable range of radial distances. Moreover, it was noted that F(r,θ) data should be obtained such that bilinear interpolation between various data points produces errors of less than 2%.

**Figure 1:**
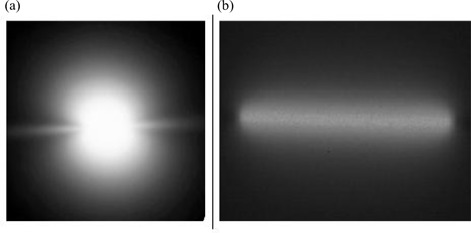
Auto radiographs of (a) a conventional seed‐type ${}^{103}Pd$ source and (b) a RadioCoil ${}^{103}Pd$ linear source (RadioMed Corporation, Tyngsboro, MA) 5.0‐cm long.

Dose distributions around brachytherapy sources with active lengths of 1.0 cm or less are nearly spherical [Fig. 1(A)]. The polar coordinate system is therefore a logical choice in the TG‐43 and TG‐43U1 recommendations for those sources.^(^ ^1^ ^,^ ^2^ ^)^ However, distribution shape has not been fully explored for elongated brachytherapy sources—that is, for those with active lengths greater than 1.0 cm. This lack of information is a hindrance for clinical application of elongated sources such as the recently introduced RadioCoil ${}^{103}Pd$ sources by RadioMed Corporation (Tyngsboro, MA). These sources are available in active lengths ranging from 1.0 cm to 6.0 cm, in 1.0‐cm steps.

In a separate investigation, we evaluated the use of TG‐43U1‐recommended parameters in a polar coordinate system for dosimetric characterization of a RadioCoil ${}^{103}Pd$ source 5.0‐cm long.^(11)^ The results indicated that use of the TG‐43U1 recommendations leads to discrepancies of up to 30% as compared with the Monte Carlo–simulated data. Those differences were attributed to the limited data points for the 2D anisotropy function and the inadequacy of the linear interpolation technique for dose distribution around an elongated source based on these limited data. The discrepancies were reduced to about 10% with the use of smaller radial increments for F(r,θ), but the TG‐43U1‐recommended 2% error could not be reached using a reasonable number of radial increments. Fig. 1(B) shows that the pattern of radiation distribution around an elongated brachytherapy source is not spherical. Hence, the use of a polar coordinate–based parameterization may not be the most effective system for implementing such sources. A different approach may therefore be needed to accurately calculate dose distributions around elongated brachytherapy sources.

**Figure 2:**
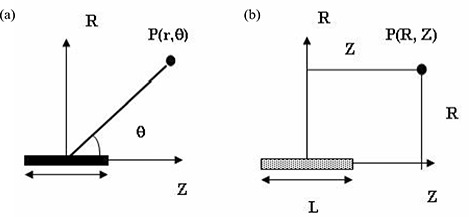
Coordinate system used for brachytherapy dosimetry calculations in (a) polar and (b) cylindrical coordinate systems.

In 2001, Schaart et al.^(12)^ explained that a straightforward application of the TG‐43 formalism to calculate the dose distribution around intravascular line sources, as proposed by TG‐60, may be difficult. They concluded that such an application would be even more difficult for line sources emitting low‐energy photons or beta particles. To resolve the limitations, they recommended the use of a formalism based on cylindrical coordinates. Similarly, in an independent investigation, Patel et al.^(^ ^13^ ^,^ ^14^ ^)^ suggested the use of a cylindrical coordinate–based TG‐43 formalism for dose calculations at short distances relative to a linear intravascular ${}^{192}Ir$ source. With some modifications, Chiu‐Tsao et al.^(15)^ implemented a cylindrical coordinate–based formalism for dose calculations around beta‐emitting intravascular brachytherapy sources. The results of the foregoing investigations indicate that the dose calculation formalism based on a cylindrical coordinate system is more suitable for dosimetry around elongated brachytherapy sources than is a polar coordinate system. Appendix A outlines a comparison between the TG‐43U1 formalism in the polar and cylindrical coordinate systems. Fig. 2(A,B) shows the coordinate system used for brachytherapy source dosimetry calculations in the polar and cylindrical coordinate systems respectively.

In the present work, we investigated the dosimetric characteristics of RadioCoil ${}^{103}Pd$ sources ranging in length from 1.0 cm to 6.0 cm, but for brevity, we present the data for the sources 1.0‐cm, 3.0‐cm, and 5.0‐cm long only. All parameters were determined using the cylindrical coordinate–based TG‐43U1 formalism.

In addition, we investigated the advantages of cylindrical over polar coordinates for dose calculations around elongated brachytherapy sources. Further, to reduce the amount of dosimetric data for treatment planning with such sources, we investigated the validity of the linear segmented source (LSS) model^(16)^ used in combination with the cylindrical coordinate–based formalism. We used the polar coordinate–based parameters published by Dini et al.^(17)^ and the values obtained using the cylindrical coordinate–based parameters to compare the calculated dose profiles.

## II. MATERIAL AND METHODS

### A. Monte Carlo calculation

In the present investigations, we used the MCNP5^(18)^ Monte Carlo code to determine the cylindrical coordinate–based TG‐43U1 dosimetric parameters for elongated (active length greater than 1 cm) RadioCoil ${}^{103}Pd$ sources. Dosimetric parameters and dose profiles around RadioCoil ${}^{103}Pd$ sources 1.0‐cm, 3.0‐cm, and 5.0‐cm long are presented here. The general‐purpose three‐dimensional radiation transport MCNP5 Monte Carlo code is designed to simulate coupled neutron, photon, and electron transport through homogeneous and heterogeneous media.

Melhus and Rivard demonstrated that the use of the *F4 tally with the $\mu_{en}/\rho$ from the National Institute of Standards and Technology (NIST) is in excellent agreement (within 0.1%) with the data obtained using the F6 tally, along with the inherent $\mu_{en}/\rho$ in the MCNP5 code for an energy range of 15 KeV to 1.5 MeV.^(19)^ Therefore, in the present investigations, the *F4 tally was used to determine the dose rate distribution around RadioCoil ${}^{103}Pd$ brachytherapy sources. The *F4 tally allows for the calculation of average photon fluence over the tally cell in units of ${MeV\;cm}^{-2}\;{photon}^{-1}$.^(18)^ However, the result can be directly converted to dose in units of ${MeV\;g}^{-1}\;{photon}^{-1}$ by incorporating the updated energy‐dependent mass–energy absorption coefficients $\left({cm}^{2}/g\right)$ into the simulation. Furthermore, we converted dose units of MeV/g per photon to ${Gy\;h}^{-1}\;U^{-1}$ by using the tally multiplier (FMn) card.^(^ ^18^ ^–^ ^20^ ^)^ The MCNP5 Monte Carlo code uses a default photon cross section library, p04, from the National Nuclear Data Center's ENDF/B‐VI.^(21)^ The mass absorption coefficients of Hubbell and Seltzer^(22)^ distributed by NIST were used to obtain absorbed dose from energy flux. For the Monte Carlo simulations, the photon spectrum of ${}^{103}Pd$ was taken from the TG‐43UI report.^(2)^ In those simulations, a 5 keV cut‐off energy was used for photons. That cut‐off is consistent with the NIST 1999 air kerma strength standard, in which an aluminum foil was used to filter the photons with energies below 5 keV in the wide‐angle free‐air chamber.^(23)^

**Figure 3:**
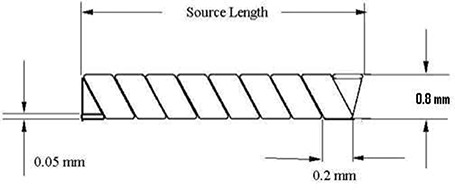
Schematic of the new RadioCoil ${}^{103}Pd$ brachytherapy source (RadioMed Corporation, Tyngsboro, MA).

Fig. 3 shows a schematic of the new RadioCoil ${}^{103}Pd$ source design used in the present investigations.^(^ ^9^ ^,^ ^17^ ^)^ In this source design, high‐purity rhodium ribbon is activated in a cyclotron to produce radioactive palladium‐103, which is then turned into a dense helix. In the Monte Carlo simulations, the geometry of the source was modeled as a cylindrical rhodium shell of 0.05 mm thickness, assuming that the effect of the helical structure of the source on dose distribution is negligible. The ${}^{103}Pd$ activity was modeled as uniformly distributed to 20 μm depth. The Monte Carlo simulations were performed by virtual placement of the source centers at the center of a spherical liquid water phantom 50.0 cm in diameter. Dose values around the source were calculated in circular tori tally cells with a cross‐sectional diameter of 1 mm and variable major radii. In addition, the dose distribution on the longitudinal axis of the sources was calculated using spherical tally cells 0.8 mm in diameter. The densities and chemical composition of the liquid water and the air used in these simulations were obtained from the TG‐43U1 report.^(2)^ The densities of ${}^{103}Pd$ and ${}^{103}Rh-\;\;\;-12.02\;g/{cm}^{3}$ and $12.41\;g/{cm}^{3}$ respectively—were obtained from the NIST web site.^(22)^

The simulations for dose rate constant, radial dose function, and 2D anisotropy function were performed for up to $8\times 10^{7}$ starting particle histories in liquid water. However, for determination of dose rates on the longitudinal axis of the sources, the simulations were performed for up to $2.4\times 10^{9}$ starting particle histories. The larger histories provided a statistical fluctuation of less than 5% for the points falling within 3 cm beyond the active length of the sources. The air kerma strengths of the sources were calculated in a spherical void phantom 100.0 cm in diameter with tally cells composed of dry air for $2\times 10^{7}$ starting particle histories. For those determinations, the air kerma rates were first calculated along the transverse bisector of each source length, at radial distances ranging from 0.5 cm to 35.0 cm in 0.5‐cm increments. The propagation of errors in these Monte Carlo simulations was estimated in the same fashion as described in our previous publication.^(17)^ From these simulations, the total errors at radial distances of 1.0 cm and 5.0 cm were found to be 2.6% and 4.3% respectively. Variation of the air kerma rates, multiplied by the square of the corresponding radial distances, was less than 1% at radial distances greater than 3L, L being the active length of the source. The product of the simulated air kerma rate at a radial distance of 5L and the square of the corresponding radius was therefore chosen as the air kerma strength of the source.

### B. Cylindrical coordinate–based TG‐43U1 dosimetric parameters

The next two subsections (II.C and II.D) describe the method of determination of the dosimetric characteristics of RadioCoil ${}^{103}Pd$ sources 1.0‐cm, 3.0‐cm, and 5.0‐cm long, in water, using the cylindrical coordinate–based TG‐43U1 formalism, as shown in Appendix A. To validate the source geometry in the Monte Carlo simulations, the values of the dose rate constant and the radial dose function from the present project were compared with the corresponding data obtained by Dini et al.^(17)^ It should be noted that the data presented by Dini et al.^(17)^ were validated by experimental data obtained using a thermoluminescence dosimetry (TLD) technique.

The dose rate constants for the RadioCoil ${}^{103}Pd$ sources were calculated as the ratio of the simulated dose rate at the reference point (that is, R=1.0 cm, Z=0) to the simulated air kerma strength (equation A‐6). The radial dose functions of the sources were calculated using equation A‐8. These calculations were performed for radial distances ranging from 0.2 cm to 1.0 cm in 0.2‐cm increments and distances ranging from 1.0 cm to 7.0 cm in 0.5‐cm increments. The 2D anisotropy functions, F(R,Z), of the sources were calculated in the cylindrical coordinate system (equation A‐14). The parameters were obtained for points with radial (R) distances ranging from 0.2 cm to 1.0 cm in 0.2‐cm increments and from 1.0 cm to 3.0 cm in 0.5‐cm increments. The Z coordinates ranged from 0.0 cm to 3.6 cm in 0.2‐cm increments. It should be noted that the $g_{L}(R)$ and F(R,Z) for the sources were determined using the linear source approximation. In those calculations, the effective length of each source was assumed to be the same as its physical length, and the geometry functions were obtained using equation A‐10 for points with R values greater than 0.

It should be noted that equations A‐10 and A‐14 (for the geometry function and the 2D anisotropy function respectively) present singularities for R=0 for points falling on the longitudinal axis of the source. Equation A‐12 has been extracted from A‐10 using l'Hôpital's rule to resolve the singularity in the geometry function. However, the singularities for the 2D anisotropy function arise from the fact that the value of the dose rate in the denominator of equation A‐14 is the dose rate at the center of the active length of the source (R=0, $Z_{o}=0$), which cannot be determined using experimental or theoretical models. As an intermediate solution, the tabulated dose rate values for the points falling on the longitudinal axis (located outside of the active length) of an elongated source have been provided for treatment planning with such sources.

### C. Cylindrical as compared with polar coordinate–based TG‐43U1 dose profile

In this subsection, the advantages of using the cylindrical over the polar coordinate–based TG‐43U1 formalism and parameters for dose calculations around elongated RadioCoil ${}^{103}Pd$ brachytherapy sources are evaluated. For these evaluations, the Monte Carlo–simulated dose profiles around a RadioCoil ${}^{103}Pd$ source 5.0‐cm long were compared with the data calculated using both polar and cylindrical coordinate–based TG‐43U1 parameters. Dose profiles were obtained along the lines parallel to the longitudinal axis of the source, with radial distances of R=0.5 cm, 0.9 cm, and 1.25 cm. For each line, dose values were calculated at several points with Z coordinates ranging from 0 cm to 3.6 cm. These calculation points were selected to create a realistic approach for dose calculations around the elongated sources. Bilinear interpolation techniques were used to extract the 2D anisotropy functions from both the cylindrical and polar coordinate–based parameters. In those calculations, the polar coordinate–based TG‐43U1 parameters were obtained from data published by Dini et al.^(17)^ The calculations were performed using Microsoft Excel 2003 installed on a Windows XP operating system.

### D. Application of the LSS model for treatment planning with cylindrical coordinate–based TG‐43U1 parameters

Implantation with linear sources may involve various source lengths for the required dose coverage within the implanted volume. Dosimetry for patients implanted with multiple source lengths demands that dosimetric characteristics be available in the treatment planning system for each source length. In an earlier project, we introduced the LSS model as an interim solution for treatment planning with elongated low‐energy brachytherapy sources.^(16)^ We demonstrated that the LSS model with the polar coordinate–based TG‐43U1 formalism re‐produces (±4%) Monte Carlo–simulated values for the points bounded within the active length of the source. However, outside of the boundary, differences of up to 14% have been observed for a source 3.0‐cm long. The current work examined the accuracy of the LSS model for dose calculation around the elongated sources using cylindrical coordinate–based TG‐43U1 parameters.

The LSS model was used to calculate dose profiles around RadioCoil ${}^{103}Pd$ sources 3.0 cm and 5.0 cm in length. In those calculations, the elongated source was replaced by a series of source segments each 1.0‐cm long, arranged in a linear fashion. The Monte Carlo–simulated dosimetric parameters of the source 1.0‐cm long in the cylindrical coordinate system were used to calculate dose profiles around a RadioCoil ${}^{103}Pd$ source 5.0‐cm long at radial distances of 0.5 cm, 0.9 cm, and 1.25 cm. The success of the LSS model with the cylindrical coordinate–based parameters will allow dosimetric parameterization of the smallest source segment (1.0 cm) to be used for dose calculations in implantations using various source lengths. That approach will not only ease the dosimetric evaluations of the sources, but will also reduce the collection and entry of data into the treatment planning system.

## III. RESULTS

Cylindrical coordinate–based TG‐43U1 dosimetric parameters (dose rate constant, radial dose function, 2D anisotropy function) of RadioCoil ${}^{103}Pd$ brachytherapy sources 1.0‐cm and 5.0‐cm long were determined using the Monte Carlo simulation technique. The results of those investigations show that the dose rate constants of the those sources in liquid water are $0.603\pm 0.016\;cGy\;h^{-1}U^{-1}$ and $0.236\pm 0.006\;cGy\;h^{-1}U^{-1}$ respectively. Table 1 compares the dose rate constants of those sources in the cylindrical coordinate system with the data in the polar coordinate system published by Dini et al.^(17)^ The small differences (<0.4%) between the dose rate constants from the two separate investigations are attributed to statistical fluctuation in the Monte Carlo simulations and the rounding of numbers during the data analysis.

**Figure 4:**
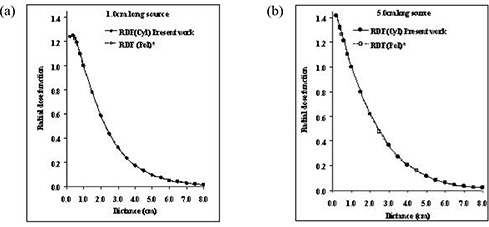
Comparison of the Monte Carlo–simulated radial dose functions (RDFs) of RadioCoil ${}^{103}Pd$ sources (RadioMed Corporation, Tyngsboro, MA), (A) 1.0‐cm and (B) 5.0‐cm long in liquid water, calculated for cylindrical and polar coordinate systems.

Fig. 4 compares the Monte Carlo–simulated radial dose function of RadioCoil ${}^{103}Pd$ sources 1.0‐cm and 5.0‐cm long obtained using the cylindrical coordinate–based TG‐43U1 formalism and the published data using the polar coordinate–based formalism.^(17)^ The results indicate excellent agreement (<1%) between the data from the cylindrical and polar coordinate–based formalisms. As described earlier, the small differences (<1%) between the data in the two separate investigations are attributed to statistical fluctuation in the Monte Carlo simulations. Table 2 presents the Monte Carlo–simulated radial dose functions for the sources. Similar results were observed for other source lengths.

**Table 1:** Comparison of dose rate constants for RadioCoil ${}^{103}Pd$ sources (RadioMed Corporation, Tyngsboro, MA) 1.0‐cm and 5.0‐cm long obtained in liquid water as determined in the present work and as published by Dini et al.^(17)^

	*Dose rate constant (cGy/h/U)*	
*Active length (cm)*	*Cylindrical coordinate (present work)*	*Polar coordinate (Dini et al*.^(17)^)
1.0	0.603±0.016	0.602±3%
5.0	0.236±0.006	0.235±3%

**Table 2:** Monte Carlo–simulated radial dose function, $g_{cyl}$ (R), of RadioCoil ${}^{103}Pd$ sources (RadioMed Corporation, Tyngsboro, MA) 1.0‐cm and 5.0‐cm long determined in liquid water using the cylindrical coordinate system

	$g_{cyl}$ *(R) for source length*	
*R (cm)*	*1.0 cm*	*5.0 cm*
0.2	1.24	1.418
0.4	1.25	1.317
0.6	1.191	1.211
0.8	1.097	1.103
1.0	1.000	1.000
1.5	0.780	0.797
2.0	0.585	0.619
2.5	0.436	0.474
3.0	0.323	0.364
3.5	0.237	0.27
4.0	0.174	0.205
4.5	0.131	—
5.0	0.096	0.115
6.0	0.052	0.062
7.0	0.029	0.031

**Figure 5:**
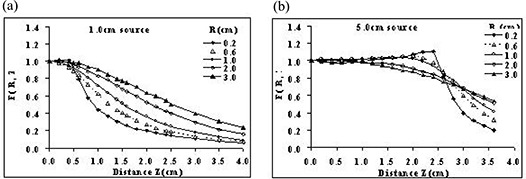
Graphical representation of the two‐dimensional anisotropy function F(R, Z) for RadioCoil ${}^{103}Pd$ sources (RadioMed Corporation, Tyngsboro, MA) (a) 1.0‐cm and (b) 5.0‐cm long, determined in liquid water using the cylindrical coordinate system.

Tables 3 and 4 respectively present the Monte Carlo–simulated F(R, Z) for RadioCoil ${}^{103}Pd$ sources 1.0 cm and 5.0 cm in length. In addition, Fig. 5(A) shows a graphical representation of F(R, Z) as a function of Z for radial distances of 0.2 cm, 0.6 cm, 1.0 cm, 2.0 cm, and 3.0 cm, for a source 1.0‐cm long. Similarly, Fig. 5(B) shows F(R, Z) for a source 5.0‐cm long. Those figures suggest that the 2D anisotropy function for these source lengths can be divided into two zones:

- the region bounded by the active length of the source, and
- the region outside the active length of the source.

**Table 3:** Two‐dimensional anisotropy function, F(R, Z), of a RadioCoil ${}^{103}Pd$ source (RadioMed Corporation, Tyngsboro, MA) 1.0‐cm long determined in liquid water using the cylindrical coordinate system

	*Radial distance R (cm)*	
*Z (cm)*	*0.20*	*0.40*	*0.60*	*0.80*	*1.00*	*1.50*	*2.00*	*2.50*	*3.00*
0.00	1.000	1.000	1.000	1.000	1.000	1.000	1.000	1.000	1.000
0.20	1.013	0.990	0.982	0.986	0.991	0.987	1.001	0.998	1.004
0.40	1.012	0.942	0.932	0.940	0.947	0.959	0.979	0.989	0.996
0.80	1.689	1.276	1.131	1.073	1.039	0.989	0.989	0.983	1.010
1.00	1.006	1.023	0.994	0.980	0.972	0.941	0.948	0.967	0.968
1.20	0.674	0.780	0.831	0.862	0.870	0.884	0.901	0.918	0.943
1.40	0.516	0.609	0.674	0.729	0.770	0.820	0.857	0.870	0.891
1.60	0.404	0.483	0.555	0.617	0.667	0.728	0.785	0.812	0.839
2.00	0.416	0.482	0.568	0.631	0.671	0.739	0.787	0.820	0.845
2.20	0.333	0.397	0.465	0.520	0.575	0.656	0.723	0.765	0.793
2.40	0.295	0.334	0.385	0.435	0.491	0.580	0.638	0.684	0.714
2.60	0.249	0.280	0.320	0.375	0.414	0.498	0.578	0.624	0.679
2.80	0.213	0.236	0.268	0.313	0.354	0.439	0.513	0.569	0.621
2.60	0.211	0.176	0.268	0.307	0.344	0.435	0.503	0.563	0.591
2.80	0.126	0.106	0.150	0.175	0.202	0.261	0.319	0.378	0.424
3.00	0.078	0.063	0.090	0.107	0.120	0.155	0.202	0.252	0.296
3.20	0.049	0.042	0.061	0.062	0.071	0.093	0.136	0.169	0.205
3.40	0.031	0.000	0.000	0.000	0.000	0.001	0.004	0.011	0.023
3.60	0.018	0.000	0.000	0.000	0.000	0.001	0.002	0.006	0.013

**Table 4:** Two‐dimensional anisotropy function, F(R, Z), of a RadioCoil ${}^{103}Pd$ source (RadioMed Corporation, Tyngsboro, MA) 5.0‐cm long determined in liquid water using the cylindrical coordinate system

	*Radial distance R (cm)*	
*Z (cm)*	*0.2*	*0.4*	*0.6*	*0.8*	*1.0*	*1.5*	*2.0*	*2.5*	*3.0*
0.00	1.000	1.000	1.000	1.000	1.000	1.000	1.000	1.000	1.000
0.20	1.005	1.003	1.000	1.006	1.014	1.000	0.991	0.983	0.992
0.40	1.005	0.996	1.002	1.004	1.010	1.002	0.986	0.995	0.973
0.60	1.004	1.000	0.998	1.008	1.014	0.993	0.994	0.999	0.973
0.80	1.005	1.004	1.013	1.015	1.019	0.999	0.989	1.007	0.986
1.00	1.011	1.008	1.012	1.022	1.019	1.009	0.986	0.986	0.983
1.20	1.009	1.024	1.032	1.025	1.030	1.001	0.977	0.974	0.968
1.40	1.023	1.022	1.031	1.041	1.035	0.993	0.971	0.972	0.941
1.60	1.028	1.046	1.046	1.037	1.037	0.993	0.959	0.941	0.915
1.80	1.040	1.053	1.053	1.049	1.039	0.974	0.938	0.923	0.883
2.00	1.070	1.070	1.051	1.041	1.020	0.945	0.910	0.868	0.874
2.20	1.100	1.079	1.037	1.013	0.992	0.923	0.872	0.862	0.835
2.40	1.107	1.023	0.978	0.956	0.938	0.884	0.846	0.826	0.809
2.60	0.824	0.871	0.874	0.863	0.858	0.824	0.796	0.767	0.757
2.80	0.560	0.687	0.730	0.763	0.773	0.752	0.735	0.718	0.724
3.00	0.394	0.527	0.597	0.633	0.674	0.679	0.683	0.694	0.674
3.20	0.313	0.400	0.490	0.539	0.569	0.613	0.616	0.622	0.630
3.40	0.251	0.322	0.392	0.449	0.485	0.527	0.562	0.574	0.585
3.60	0.199	0.271	0.320	0.376	0.415	0.474	0.511	0.519	0.537

The variation of the 2D anisotropy function within the active region of the source is greater for larger radial distances (R). However, for the regions outside the active length of the source, the dose gradient is larger at shorter radial distances.

**Figure 6:**
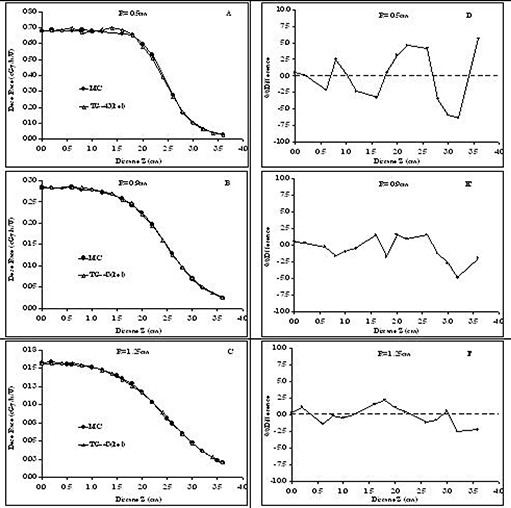
Left‐hand panels: Comparison of Monte Carlo (MC)–simulated and analytically calculated (TG‐43U1) dose profiles at radial distances of (A) 0.5 cm, (B) 0.9 cm, and (C) 1.25 cm from a RadioCoil ${}^{103}Pd$ source (RadioMed Corporation, Tyngsboro, MA) 5.0‐cm long. Analytically calculated values are obtained using TG‐43U1 parameters in the polar coordinate system recommended by Dini et al.^(17)^ Right‐hand panels: Percentage differences between MC–simulated and analytically calculated values at radial distances of (A) 0.5 cm, (B) 0.9 cm, and (C) 1.25 cm from a RadioCoil ${}^{103}Pd$ source 5.0‐cm long.

Fig. 6(A,B,C) compares the Monte Carlo‐simulated and analytically calculated dose profiles around a RadioCoil ${}^{103}Pd$ source 5.0‐cm long at radial distances of 0.5 cm, 0.9 cm, and 1.25 cm. The analytical calculations for a RadioCoil ${}^{103}Pd$ source 5.0‐cm long were performed using the TG‐43U1 parameters with polar coordinates obtained from Dini et al.^(17)^ Fig. 6(D,E,F) presents the corresponding percentage differences between the Monte Carlo‐simulated data and the analytically calculated values. The comparisons indicate differences of up to 7% between the two data sets for radial distances (R) less than 1.0 cm. However, the differences decline with increasing radial distance. Similar results were observed for the other source lengths.

**Figure 7:**
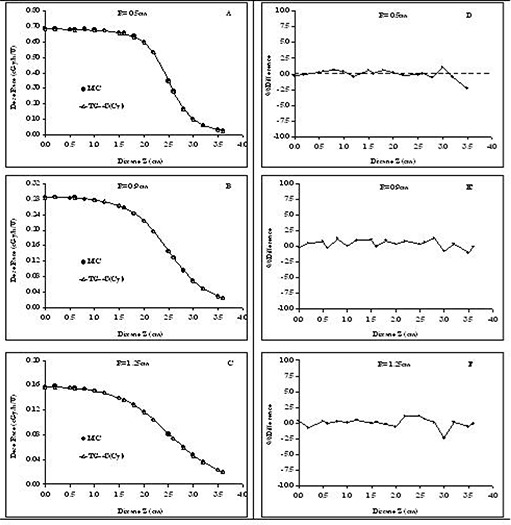
Left‐hand panels: Comparison of Monte Carlo (MC)–simulated and analytically calculated (TG‐43U1) dose profiles at radial distances of 0.5 cm (A) 0.5 cm, (B) 0.9 cm, and (C) 1.25 cm from a RadioCoil ${}^{103}Pd$ source (RadioMed Corporation, Tyngsboro, MA) 5.0‐cm long. Analytically calculated values are obtained using TG‐43U1 parameters in the cylindrical coordinate system. Right‐hand panels: Percentage differences between MC–simulated and analytically calculated values at radial distances of (A) 0.5 cm, (B) 0.9 cm, and (C) 1.25 cm from a RadioCoil ${}^{103}Pd$ source 5.0‐cm long.

Fig. 7(A,B,C) compares the Monte Carlo–simulated dose profiles and analytically calculated data using cylindrical coordinate–based parameters at radial distances of 0.5 cm, 0.9 cm, and 1.25 cm for a RadioCoil ${}^{103}Pd$ source 5.0‐cm long. Fig. 7(D,E,F) presents the percentage difference between the two data sets. Excellent agreement (±2%)) was observed between the Monte Carlo‐simulated and analytically calculated dose profiles. Similar accuracy was observed for the cylindrical coordinate‐based parameters for source lengths ranging from 1.0 cm to 6.0 cm.

**Figure 8:**
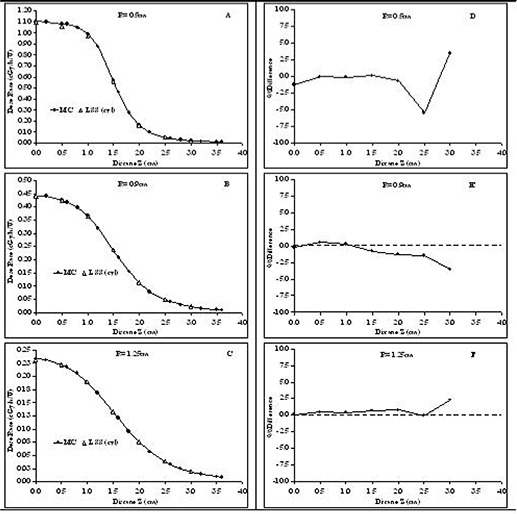
Left‐hand panels: Comparison of Monte Carlo (MC)–simulated and analytically calculated (LSS) dose profiles at radial distances of (A) 0.5 cm, (B) 0.9 cm, and (C) 1.25 cm from a RadioCoil ${}^{103}Pd$ source (RadioMed Corporation, Tyngsboro, MA) 3.0‐cm long. Analytically calculated values are obtained using the linear segmented source (LSS) model and TG‐43U1 parameters for a source 1.0‐cm long in the cylindrical coordinate system. Right‐hand panels: Percentage differences between MC–simulated and analytically calculated dose profiles at radial distances of (D) 0.5 cm, (E) 0.9 cm, and (F) 1.25 cm from a RadioCoil ${}^{103}Pd$ source 3.0‐cm long.

**Figure 9:**
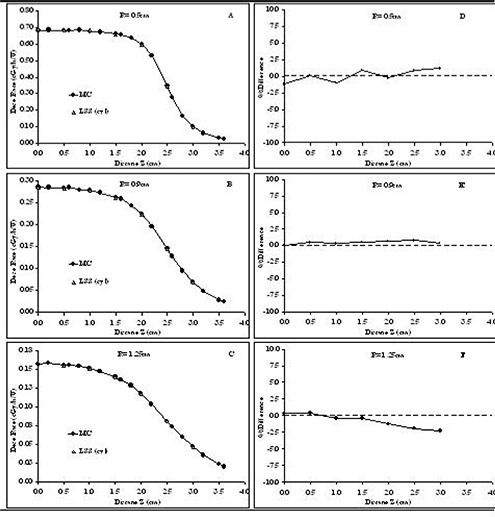
Left‐hand panels: Comparison between Monte Carlo (MC)–simulated and analytically calculated dose profiles at radial distances of (A) 0.5 cm, (B) 0.9 cm, and (C) 1.25 cm from a RadioCoil ${}^{103}Pd$ source (RadioMed Corporation, Tyngsboro, MA) 5.0‐cm long. Analytically calculated values are obtained using the linear segmented source (LSS) model and TG‐43U1 parameters for a source 1.0‐cm long in the cylindrical coordinate system. Right‐hand panels: Percentage differences between MC–simulated and analytically calculated dose profiles at radial distances of (D) 0.5 cm, (E) 0.9 cm, and (F) 1.25 cm from a RadioCoil ${}^{103}Pd$ source 5.0‐cm long.

Figs. 8 and 9 respectively compare the Monte Carlo‐simulated dose profiles around RadioCoil ${}^{103}Pd$ sources 3.0‐cm and 5.0‐cm long with the values calculated using the LSS model. The calculations using the LSS model were based on TG‐43U1 parameters with cylindrical coordinates for a RadioCoil ${}^{103}Pd$ source 1.0‐cm long. Fig. 8(D,E,F) shows the percentage difference between the Monte Carlo–simulated and the LSS model‐calculated dose profiles for a source 3.0‐cm long. Fig. 9(D,E,F) demonstrates a similar comparison for a source 5.0‐cm long. The results indicate that, for all of the points with Z≤L / 2+0.52 cm, the Monte Carlo–simulated and analytically calculated dose profiles show excellent agreement (±2.5%). However, for Z>L //2+0.5, the differences increase to ±5% because of the lower dose rate, which leads to larger statistical fluctuations in the Monte Carlo simulations.

Table 5 shows tabulated dose‐rate values $\left({cGY\;h}^{-1}U^{-1}\right)$ for dose calculations at the points falling on the longitudinal axis (that is, R=0.0) for RadioCoil ${}^{103}Pd$ sources 1.0‐cm, 3.0‐cm, and 5.0‐cm long. We used the data from the source 1.0‐cm long to examine reproduction by the LSS model of the dose rates on the longitudinal axis, beyond the tip and end of the physical source, for sources 3.0‐cm and 5.0‐cm long. Table 6 compares the Monte Carlo–simulated dose rates for RadioCoil ${}^{103}Pd$ sources 3.0‐cm and 5.0‐cm long with the values calculated using the LSS model and the data for the source 1.0‐cm long. The results indicate good agreement (within 5%) for the points within Z≤L / 2+1.0 cm from the end of the active length. However, at larger distances, an increase in the differences between the two data sets (up to 10%) was observed. That increase could be attributed to the larger statistical fluctuation of the Monte Carlo simulations.

**Table 5:** Monte Carlo–simulated dose rates $\left({cGY\;h}^{-1}U^{-1}\right)$ on the longitudinal axis (that is, R=0.0) of RadioCoil ${}^{103}Pd$ sources (RadioMed Corporation, Tyngsboro, MA) 1.0‐cm, 3.0‐cm, and 5.0‐cm long^a^

	*Active length of source (cm)*		
*Z (cm)*	*1.0*	*3.0*	*5.0*
0.6	5.71E+00	—	—
0.8	8.31E−01	—	—
1.0	3.24E−01	—	—
1.2	1.72E−01	—	—
1.5	8.06E−02	—	—
1.6	7.00E−02	1.94E+00	—
1.8	4.94E−00	3.00E−01	—
2.0	3.30E−02	1.25E−01	—
2.2	2.50E−02	6.75E−02	—
2.5	1.60E−02	3.50E−02	—
2.6	1.39E−02	2.76E−02	1.18E+00
2.8	1.11E−02	2.01E−02	1.84E−01
3.0	8.37E−03	1.54E−02	7.57E−02
3.2	7.11E−03	1.13E−02	4.10E−02
3.5	4.92E−03	6.87E−03	2.16E−02
3.6	4.60E−03	2.82E−03	1.86E−02
3.8	3.67E−03	—	—
4.0	2.87E−03	—	—
4.2	2.26E−03	—	—
4.5	1.78E−03	—	—
4.6	1.53E−03	—	—
4.8	1.18E−03	—	—
5.0	9.17E−04	—	—
5.2	5.74E−04	—	—
5.5	5.86E−04	—	—
5.6	6.54E−04	—	—

a These points fall outside of the active length of the sources.

**Table 6:** Comparison of Monte Carlo (MC)–simulated dose rates $\left({cGY\;h}^{-1}U^{-1}\right)$ on the longitudinal axis (that is, R=0.0) with values calculated using the linear segmented source (LSS) model, for RadioCoil ${}^{103}Pd$ sources (RadioMed Corporation, Tyngsboro, MA) 3.0‐cm and 5.0‐cm long^a^

*Z (cm)*	*Active length of the source (cm)*					
	*MC*	*3.0 LSS*	*% Diff*.	*MC*	*5.0 LSS*	*% Diff*.
1.6	1.94E+00	1.93E+00	−0.6		—	
1.8	3.00E−01	2.97E−01	−1		—	
2.0	1.25E−01	1.22E−01	−2.6		—	
2.2	6.75E−02	6.80E−02	0.6		—	
2.5	3.50E−02	3.38E−02	−3.4		—	
2.6	2.76E−02	2.95E−02	6.9	1.18E+00	1.16E+00	−1.7
2.8	2.01E−02	2.14E−02	6.3	1.84E−01	1.79E−01	−2.4
3.0	1.54E−02	1.48E−02	−4.1	7.57E−02	7.38E−02	−2.4
3.2	1.13E−02	1.15E−02	1.5	4.10E−02	4.13E−02	0.8
3.5	6.87E−03	7.56E−03	10	2.16E−02	2.08E−02	−3.7
3.6	6.13E−03	6.66E−03	8.6	1.86E−02	1.81E−02	−2.4

a These points fall outside of the active length of the sources.

## IV. DISCUSSION AND CONCLUSIONS

Several investigators have demonstrated the suitability of the cylindrical coordinate–based formalism for dose calculation around elongated brachytherapy sources.^(^ ^12^ ^,^ ^13^ ^)^ In the present work, the updated TG‐43U1 protocol, presented in a cylindrical coordinate system, was used for dosimetric parameterization of RadioCoil ${}^{103}Pd$ sources 1.0 cm and 5.0 cm in length. The Monte Carlo simulation technique was used to determine dose rate constants, radial dose functions, and 2D anisotropy functions of these sources in water.

As shown in Appendix A, the modified TG‐43 formalism in the cylindrical coordinate system and the corresponding parameters were selected such that the dose rate constant and radial dose functions were identical to those in the polar coordinate system.^(17)^ Fig. 4 demonstrates the concept by comparing the radial dose functions of RadioCoil ${}^{103}Pd$ sources 1.0‐cm and 5.0‐cm long in the two coordinate systems. In addition, Table 1 compares the dose rate constants for the source lengths determined earlier in the present work and the values published by Dini et al.^(17)^ using the polar coordinate system. It should be noted that, despite the identical nature of the radial dose functions for a brachytherapy source in the two coordinate systems, different values might be required for dose calculation at a given point. For example, for a dose calculation using the polar coordinate system at a given point P (3 cm, 30 degrees) relative to a RadioCoil ${}^{103}Pd$ source 5.0‐cm long, the TG‐43U1 formalism requires a value $g_{\text{pol}}.(3.0\;\text{cm})=0.364$ (Table 2). However, for dose calculation using the cylindrical coordinate system at the same point, the modified formalism requires the value $g_{cyl}$.[R=3sin⁡(30 degrees)=1.5 cm]=0.797.

Tables 3 and 4 show the cylindrical coordinate–based 2D anisotropy functions of the sources investigated earlier. Although the mathematical definitions of F(r,θ) and F(R,Z) are similar, their values are different. Fig. 5 demonstrates that the variation of F(R, Z) is minimal within the active region of the source, but significant outside of that region. These variations closely represent the variation of dose distribution around the elongated brachytherapy sources, as shown in Fig. 1(B).

One of the main advantages of cylindrical coordinate–based TG‐43U1 parameterization over that based on polar coordinates for elongated brachytherapy sources can be found by comparing the dose profiles shown in Figs. 6 and 7. The results in Fig. 7 indicate excellent agreement (within ±1% at close distances, and a maximum of 2.5% at larger distances) between the Monte Carlo–simulated dose profiles for a RadioCoil ${}^{103}Pd$ source 5.0‐cm long and the values calculated using the cylindrical coordinate parameters. However, despite the use of the 2D anisotropy functions recommended by Awan et al.,^(11)^ differences of approximately ±7% at a 5‐mm radial distance have been observed between the Monte Carlo–simulated values and the values calculated using the polar coordinate–based parameters published by Dini et al.^(17)^ (Fig. 6). Although those differences were reduced to about ±3%; at larger radial distances, the overall agreement of the data with the cylindrical coordinate–based parameters was superior.

Fig. 8 demonstrates another advantage of the cylindrical coordinate–based parameters. The results in the figure indicate that the dose profiles around RadioCoil ${}^{103}Pd$ sources 3.0 cm and 5.0 cm in length were replicated to within ±2% by the LSS model using cylindrical coordinate– based TG‐43U1 parameters for a source 1.0‐cm long. This improvement is again significant as compared with the 14% differences found with the same model, but using polar coordinate– based parameters for a source 1.0‐cm long.^(16)^ The results of these investigations indicate that the LSS model with cylindrical coordinate–based dosimetric parameters can accurately reproduce the dose distributions around elongated sources. This approach minimizes the number of data points that would be needed to perform the treatment planning for implantation using various source lengths.

**Figure 10:**
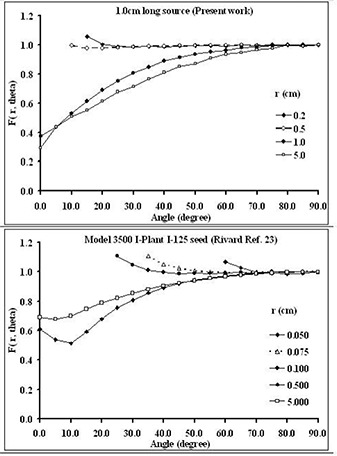
Comparison of Monte Carlo–simulated two‐dimensional anisotropy functions for 1.0‐cm (RadioCoil ${}^{103}Pd$: RadioMed Corporation, Tyngsboro, MA) and 0.5‐cm seed‐type (Model 3500 I‐Plant ${}^{125}I$) brachytherapy sources.

Interestingly, the methodologies introduced in the present work could be extended to dosimetric evaluations of the traditional seed‐type brachytherapy sources, particularly in close proximity of the source, which has clinical relevance in many cases. For example, various models of I125 and ${}^{103}Pd$ sources are being used for eye‐plaque therapy. For these treatments, the accuracy of the calculated dose to various parts of the eyeball, such as sclera, optic nerve, and macula, is crucial for the treatments. Currently, few publications are available on dosimetric evaluations of seed‐type sources at close proximity.^(24)^ Fig. 10(A) shows a rare set of dosimetric data collected for a conventional Model 3500 I‐Plant ^125^I seed.^(24)^ The results indicate that the large variations in the 2D anisotropy function of the source outside the TG‐43U1– recommended radial distances are similar to those for a RadioCoil ${}^{103}Pd$ source 1.0‐cm long.^(17)^ Significance of the data at close proximity to this model of a seed‐type source can be demonstrated by comparing the Monte Carlo–simulated F(r=0.1 cm,θ=35 degrees)=1.107 to F(r=0.5 cm,θ=35 degrees)=0.856, because TG‐43U1 recommends using the value F(0.5,θ) for 2D anisotropy functions at short radial distances. Therefore, in the absence of values at close proximity (that is, 0.1 cm), the value of the 2D anisotropy function used in the calculation would have been 0.856 rather than the Monte Carlo–simulated value of 1.107 (a difference of about 30%). Determination of the polar coordinate–based 2D anisotropy function is notably difficult at short distances (comparable to the source diameter), where most of the calculation points fall on the source itself. The loss of data points is more significant for elongated brachytherapy sources. The cylindrical coordinate–based formalism allows for calculation of the 2D anisotropy function as close as the surface of the source, and it facilitates the interpolation and extrapolation of that parameter for dose calculation purposes.

To summarize, cylindrical coordinate–based TG‐43U1‐recommended dosimetric characteristics of elongated RadioCoil ${}^{103}Pd$ sources were determined and are presented here. The advantages of these formalisms relative to the polar coordinate system have also been confirmed. As demonstrated in our findings, the cylindrical coordinate formalism significantly improves on the dosimetric evaluation of elongated sources. However, the similarity in the mathematical description of the polar coordinate–based formalism will facilitate its adoption into treatment planning systems (Appendix A). In addition, the application of the cylindrical coordinate–based TG‐43U1 formalism could be extended to dosimetric evaluations in close proximity to conventional seed‐type sources.

## ACKNOWLEDGMENT

The authors thank Dr. Lee Johnson and Jennifer Cole for their valuable scientific and editorial comments during the preparation of this manuscript. This project was partially supported by U.S. Army Medical Research under DAMD 17‐02‐1‐0242 and by RadioMed.

## Appendix

**Comparison of polar and cylindrical coordinate–based TG‐43 formalism**

Figure 2 shows schematic diagrams of dose calculation point “P” in (A) polar and (B) cylindrical coordinate systems around a linear brachytherapy source.

The relationships between the coordinates of a given point in these two systems are

(1) Z=rcos(θ)

(2) R=rsin(θ)

The main formalism for 2D dose calculation in the two coordinate systems is

(3) $$\dot{D}(r,\theta )=S_{k}\cdot \Lambda \cdot \frac{G(r,\theta )}{G(r_{o},\theta_{o})}\cdot g(r)\cdot F(r,\theta )$$

(4) $$\dot{D}(R,Z)=S_{k}\cdot \Lambda \cdot \frac{G(R,Z)}{G(R_{o},Z_{o})}\cdot g(R)\cdot F(R,Z),$$

where $r_{o}=1.0$ cm, $\theta_{o}=90$ degrees, $R_{o}=1.0$ cm, and $Z_{o}=0$ are the values of coordinates of reference points in the two systems. Dose rate constants in polar and cylindrical coordinate systems are defined in equations A‐5 and A‐6 respectively:

(5) $$\Lambda_{\text{pol}}=\frac{\dot{D}(r=1\text{cm},\theta =\pi /2)}{S_{K}}$$

(6) $$\Lambda_{\text{cyl}}=\frac{\dot{D}(R=1\text{cm},Z=0)}{S_{K}},$$

where P(r=1.0 cm, θ=90 degrees) is the same as P(R=1.0 cm, and Z=0). Therefore, the dose rate constants in the two systems are the same.

Radial dose functions in polar and cylindrical coordinate systems are presented in equations A‐7 and A‐8 respectively:

(7) $$g_{L}^{\text{pol}}(r)=\frac{\dot{D}(r,\theta_{o})G(r_{o},\theta_{o})}{\dot{D}(r_{o},\theta_{o})G(r,\theta_{o})}$$

(8) $$g_{L}^{\text{cyl}}(R)=\frac{\dot{D}(R,Z_{o})G(R_{o},Z_{o})}{\dot{D}(R_{o},Z_{o})G(R,Z_{o})}.$$

Geometry functions in polar and cylindrical coordinate systems are given as follows:

(9) $$G_{\text{pol}}(r,\theta )=\frac{\tan^{-1}[(r\cos\theta +L/2)/r\sin\theta ]-\tan^{-1}[(r\cos\theta -L/2)/r\sin\theta ]}{L.r.\sin\theta }$$

(10) $$G_{\text{cyl}}(R,Z)=\frac{\tan^{-1}[(Z+L/2)/R]-\tan^{-1}[(Z-L/2)/R]}{R.L}$$

For the points falling on the longitudinal axis of the source (that is, θ=0 in the polar, or R=0 in the cylindrical coordinate system), these equations simplify to

(11) $$G_{pol}\;(r,0)=\frac{1}{r^{2}-{\left(L/2\right)}^{2}}$$

(12) $$G_{cyl}(0,Z)=\frac{1}{Z^{2}-{\left(L/2\right)}^{2}}.$$

In the polar and cylindrical coordinate systems, the 2D anisotropy functions of brachytherapy sources are defined in equations A‐13 and A‐14 respectively:

(13) $$F_{\text{pol}}(r,\theta )=\frac{\dot{D}(r,\theta )G(r,\theta_{o})}{\dot{D}(r,\theta_{o})G(r,\theta )}$$

(14) $$F_{\text{cyl}}(R,Z)=\frac{\dot{D}(R,Z)G(R,Z_{o})}{\dot{D}(R,Z_{o})G(R,Z)}$$

Considering the relationship between the polar and cylindrical coordinate systems shown in equations A‐1 and A‐2, the geometry function, the radial dose function, and the dose rate constant can be shown to be the same. It should be noted that for the same radial distances of r=R, the values of the radial dose function in the two coordinate systems are identical. Although the main concept and formalism for the 2D anisotropy function are similar in the two coordinates systems, the values are not the same, because their normalization points are different. In polar coordinate‐based parameters, the dose rate at any angle is normalized with the dose rate at the same radial distance of “r” and θ=90. However, in cylindrical coordinate‐based parameters, the dose rate at any point is normalized to the value at same “R” and “$Z_{o}=0$.
