# Erratum: “Cylindrical coordinate‐based TG‐43U1 parameters for dose calculation around elongated brachytherapy sources”

**DOI:** 10.1002/j.1526-9914.2008.tb00001.x

**Published:** 2016-12-21

**Authors:** Shahid B Awan

**Affiliations:** ^1^ University of Kentucky Department of Radiation Medicine Lexington Kentucky U.S.A

In this erratum, we present the corrections for three typographical errors that occurred in the above noted publication on pages 123, 130 and page 138, respectively.

- The institutional affiliation for co‐author Manzoor Hussain is missing. The affiliation of Manzoor Hussain is University of the Punjab, Department of Physics, Jinnah Campus, Lahore, Pakistan. With this correction, the institutional affiliations of all the authors will be as follows:
  Shahid B. Awan,^a,b^ Sharifeh A. Dini^b^, Manzoor Hussain^a^, David Soleimani‐Meigooni^b^, and Ali S. Meigooni^b^
  University of the Punjab,^a^ Department of Physics, Jinnah Campus, Lahore, Pakistan;
  University of Kentucky,^b^ Department of Radiation Medicine, Lexington, Kentucky, U.S.A.
- Table 3 (on page 130) had a typographical error. The corrected data for 1 cm long RadioCoil P103d source is shown in the following Table. It should be noted the graphical representation of the 2D‐aniotropy is does not need any correction.
- On the legend of Fig. 10, the paper by Rivard et al. was referred to as “Rivard Ref. 23” instead of “Rivard Ref. 24”. The corrected figure caption for Fig. 10 is:

**Table 1:** Two‐dimensional anisotropy function, F(R, Z), of a RadioCoil P103d source (RadioMed Corporation, Tyngsboro, MA) 1.0‐cm long determined in liquid water using the cylindrical coordinate system.

	*Radial distance R (cm)*								
*Z (cm)*	*0.2*	*0.4*	*0.6*	*0.8*	*1*	*1.5*	*2*	*2.5*	*3*
0	1.000	1.000	1.000	1.000	1.000	1.000	1.000	1.000	1.000
0.2	1.013	0.990	0.982	0.986	0.991	0.987	1.001	0.998	1.004
0.4	1.012	0.942	0.932	0.940	0.947	0.959	0.979	0.989	0.996
0.5	—‐	0.887	0.885	0.905	0.917	0.932	0.961	0.971	0.981
0.6	0.793	0.821	0.842	0.866	0.883	0.902	0.934	0.945	0.982
0.8	0.573	0.674	0.730	0.775	0.809	0.845	0.884	0.922	0.934
1	0.438	0.542	0.619	0.680	0.717	0.784	0.831	0.868	0.905
1.2	0.364	0.445	0.515	0.580	0.636	0.722	0.785	0.817	0.849
1.4	0.302	0.368	0.435	0.498	0.554	0.639	0.716	0.758	0.796
1.5	—‐	0.340	0.405	0.463	0.514	0.607	0.677	0.733	0.775
1.6	0.259	0.306	0.373	0.429	0.474	0.570	0.648	0.707	0.753
1.8	0.218	0.264	0.317	0.364	0.414	0.508	0.593	0.656	0.703
2	0.201	0.231	0.271	0.312	0.361	0.452	0.524	0.585	0.630
2.2	0.176	0.200	0.231	0.275	0.310	0.391	0.475	0.532	0.598
2.4	0.155	0.173	0.199	0.234	0.269	0.348	0.423	0.485	0.545
2.5	0.145	0.163	0.187	0.218	0.248	0.328	0.397	0.462	0.501
3	0.110	0.122	0.132	0.155	0.179	0.235	0.292	0.349	0.397
3.5	0.083	0.086	0.095	0.113	0.127	0.163	0.212	0.262	0.306
4	0.061	0.066	0.075	0.077	0.087	0.112	0.160	0.196	0.233

**Figure 1:**
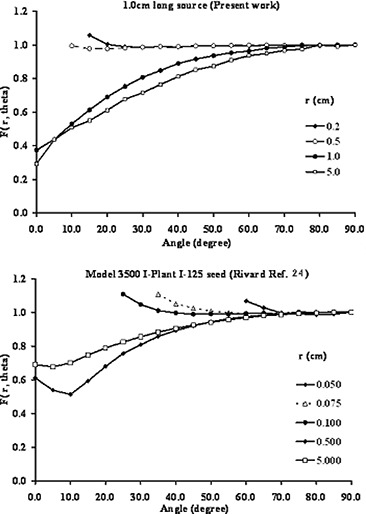
Comparison between the Monte Carlo simulated 2D anisotropy function of a 1.0 cm (RadioCoil P103d) and 0.5 cm seed type (Model 3500 I‐Plant I‐125) brachytherapy sources.

